# Supplementary material for: Complex genotype-phenotype relationships shape the response to treatment of down syndrome childhood acute lymphoblastic leukaemia
Source: Sci Rep. 2025 Nov 25;15:42018. doi: 10.1038/s41598-025-28779-9 (PMC12658182; doi:10.1038/s41598-025-28779-9)
Supplement: Supplementary file 1 — Supplementary Material 1 [file 41598_2025_28779_MOESM1_ESM.pdf]

Supplementary Methods

Model Description

1 Assumptions

The following assumptions are made:

1. There occur no mutations in the mice.
2. Traits detected in the primary transplant but not in the bulk are explained by small clones that have been present but not detected in the patient sample.
3. Traits detected in the secondary transplant but not in the bulk/primary transplant are explained by small clones that have been present but not detected in the patient sample/primary transplant.
4. The traits B1, B2, B3 always coexist with trait A.
5. No other assumptions on coexistence of traits are imposed. The clones that are selected by the model are compared to the clones deduced based on sequencing data.

2 Model

Systems of linear equations are used to check if a set of clones can explain measurements. The following traits are taken into account: A, $B1$, $B2$, $B3$, C, D, E, F. Each clone can harbor an arbitrary combination of these traits. The only constraint imposed on the combination of the traits is that $B1$, $B2$, $B3$ have to coexist with A.

Germ line contamination can be taken into account by introducing a clone harboring none of the traits A, $B1$, $B2$, $B3$, C, D, E, F.

Consider a given subset S of n clones which are numbered with indices from 1 to n. Denote by x_i the frequency of clone i in a given sample. The frequencies x_i can take values between 0% and 100% ($x_i \in [0\%,\ 100\%]$). For a given sample the frequencies x_i have to sum up to 100%, $\sum_{i=1}^{100} x_i = 100\%$. Assume that $i_1, \ \ldots, \ i_k$ are exactly the indices of the clones harboring trait Y. The total fraction of cells with trait Y, f_Y, then calculates as $f_y = x_{i_1} + \cdots + x_{i_k}$.

Denote by f_A the fraction of cells harboring trait A in a given sample, by f_{B1} the fraction of cells harboring trait $B1$ etc. The fractions are measured in per cent (%). Set $\mathbf{f} = (f_A, f_{B1}, f_{B2}, f_{B3}, f_C, f_D, f_E, f_F, 100)^T$. The vector $\mathbf{f}$ contains the measurements. The last component of $\mathbf{f}$ is needed to guarantee that the sum of all observed cells corresponds to 100%. Define the matrix M_S with entries m_{ij} ($j = 1, \cdots, n$; $i = 1, \cdots, 9$) as follows:

$$
\begin{aligned}
m_{1j} &= \begin{cases} 1 & \text{if clone } j \text{ has trait } A \\ 0 & \text{otherwise} \end{cases} \\
m_{2j} &= \begin{cases} 1 & \text{if clone } j \text{ has trait } B1 \\ 0 & \text{otherwise} \end{cases} \\
& \vdots \\
m_{8j} &= \begin{cases} 1 & \text{if clone } j \text{ has trait } F \\ 0 & \text{otherwise} \end{cases} \\
m_{9j} &= 1
\end{aligned}
$$

The last row of the matrix assures that all cells sum up to 100%. To determine whether there exists a combination of all clones from subset S that reproduces the measurements $\mathbf{f}$, we have to establish the existence of a positive solution of the linear system

$$M\mathbf{x} = \mathbf{f}. \tag{1}$$

The vector $\mathbf{x} = (x_1, \ldots x_n)^T$ contains the frequencies of clones 1 to n, as described above. The values x_i are between 0% and 100%.

3 Computational approach

The assumptions made above lead to a maximal number of 80 different clones, if germ line contamination is considered, otherwise the maximal number of different clones is 79. The number of different (unordered) subsets out of 80 different clones is given by

$$\sum_{k=1}^{80} \binom{80}{k} = 2^{80} - 1 \approx 1.2 \cdot 10^{24}.$$

In case of 79 clones it calculates as $\approx 6.0 \cdot 10^{23}$.

In a first step, we checked for each of the samples which subsets of the total 80 (79) clones can reproduce the measurements. Each mouse corresponds to one sample and the patient bulk cells correspond to one sample. Since none of the samples contains all traits, the number of possible clones can be reduced a priori: If a given sample does not harbor trait X, it can contain only clones that do not harbor trait X. In a second step, for all possible combinations of the clones remaining after the a priori reduction, the existence of positive solutions of system (1) was investigated. All possible subsets of a given set of clones were obtained using the function *combinations* from the Python software module *itertools* (Python Version 2.7.6, Python Software Foundation, Delaware).

To decide whether system (1) has a positive solution, we implemented the algorithm described in [1]. If there exists a positive solution, the algorithm provides an example solution. Using standard linear algebra, we calculated the dimension of the solution space. In cases where the solution is not unique, we used linear programming to determine maximal and minimal fractions of the considered clones. This was done using the function *linprog* from the Matlab software package (Matlab, Version R2014a, The MathWorks Inc., Massachusetts), which is based on an interior point method. The maximum fraction of clone i was obtained by maximizing x_i under the constraints from equation (1) and $x_i \geq 0$ for $i = 1, \cdots, n$. Maximality of the

numerical solution was tested using the complementary slackness condition from [2] with an error tolerance of 10^{-8}. To obtain minima, we proceeded analogously.

Assumptions 2. and 3. imply that clones present in at least one sample are also present in the patient cell bulk. The composition of the patient cell bulk can, therefore, be obtained from the clones present in the individual samples. Since different combinations of clones can be used to reproduce one single measurement, the clonal composition of the patient cell bulk is not unique. Formally: Let $S_1^k, \cdots, S_{q_k}^k$ be all subsets of clones compatible with data from sample k. Denote by s the total number of samples. Each set of the type $\cup_{i=1,\cdots,s} S_{r_i}^i$ with r_i chosen from $\{1, \cdots q_i\}$ corresponds to one possible composition of the patient cell bulk.

References

[1] Dines LL. On Positive Solutions of a System of Linear Equations. *Annals of Mathematics, Second Series* Vol. 28, No. 1/4 (1926 - 1927), pp. 386-392.

[2] Griva I, Nash SG, Sofer A. *Linear and Nonlinear Optimization*, Second Edition, Siam, Phiadelphia, 2009, p. 183.
