# Supplementary material for: Complex genotype-phenotype relationships shape the response to treatment of down syndrome childhood acute lymphoblastic leukaemia
Source: Sci Rep. 2025 Nov 25;15:42018. doi: 10.1038/s41598-025-28779-9 (PMC12658182; doi:10.1038/s41598-025-28779-9)
Supplement: Supplementary file 2 — Supplementary Material 2 [file 41598_2025_28779_MOESM2_ESM.pptx]

## Slide 1
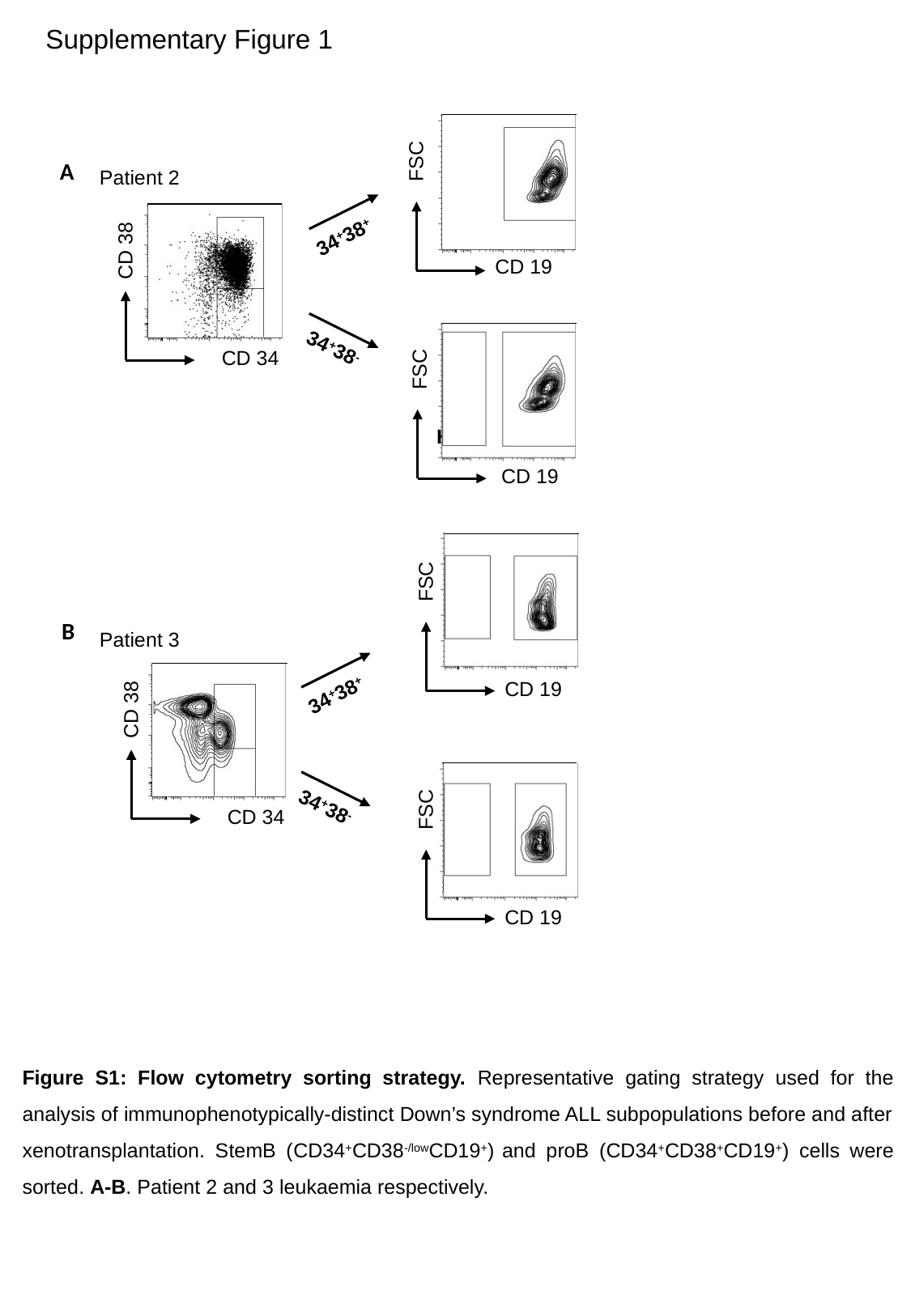

Supplementary Figure 1
FSC
A
Patient 2
ProB
34+38+
CD 38
CD 19
34+38-
CD 34
FSC
HSC
Stem/B
CD 19
FSC
B
Patient 3
ProB
CD 19
34+38+
CD 38
34+38-
FSC
CD 34
HSC
Stem/B
CD 19
Figure S1: Flow cytometry sorting strategy. Representative gating strategy used for the analysis of immunophenotypically-distinct Down’s syndrome ALL subpopulations before and after xenotransplantation. StemB (CD34+CD38-/lowCD19+) and proB (CD34+CD38+CD19+) cells were sorted. A-B. Patient 2 and 3 leukaemia respectively.

## Slide 2
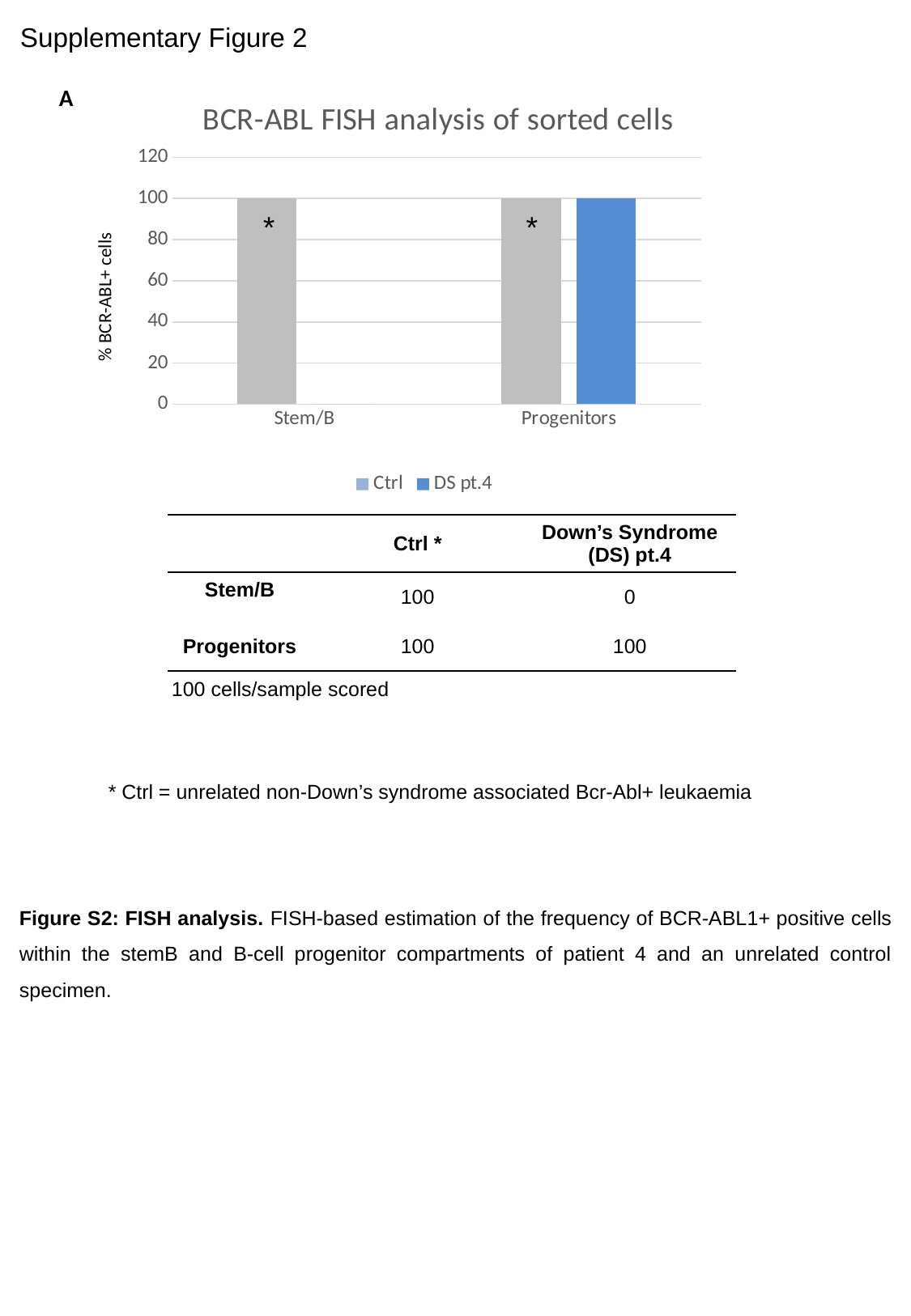

Supplementary Figure 2
A
### Chart: BCR-ABL FISH analysis of sorted cells
| Category | Ctrl | DS pt.4 |
|---|---|---|
| Stem/B | 100.0 | 0.0 |
| Progenitors | 100.0 | 100.0 |*
*
% BCR-ABL+ cells
| | Ctrl \* | Down’s Syndrome (DS) pt.4 |
| --- | --- | --- |
| Stem/B | 100 | 0 |
| Progenitors | 100 | 100 |
100 cells/sample scored
* Ctrl = unrelated non-Down’s syndrome associated Bcr-Abl+ leukaemia
Figure S2: FISH analysis. FISH-based estimation of the frequency of BCR-ABL1+ positive cells within the stemB and B-cell progenitor compartments of patient 4 and an unrelated control specimen.

## Slide 3
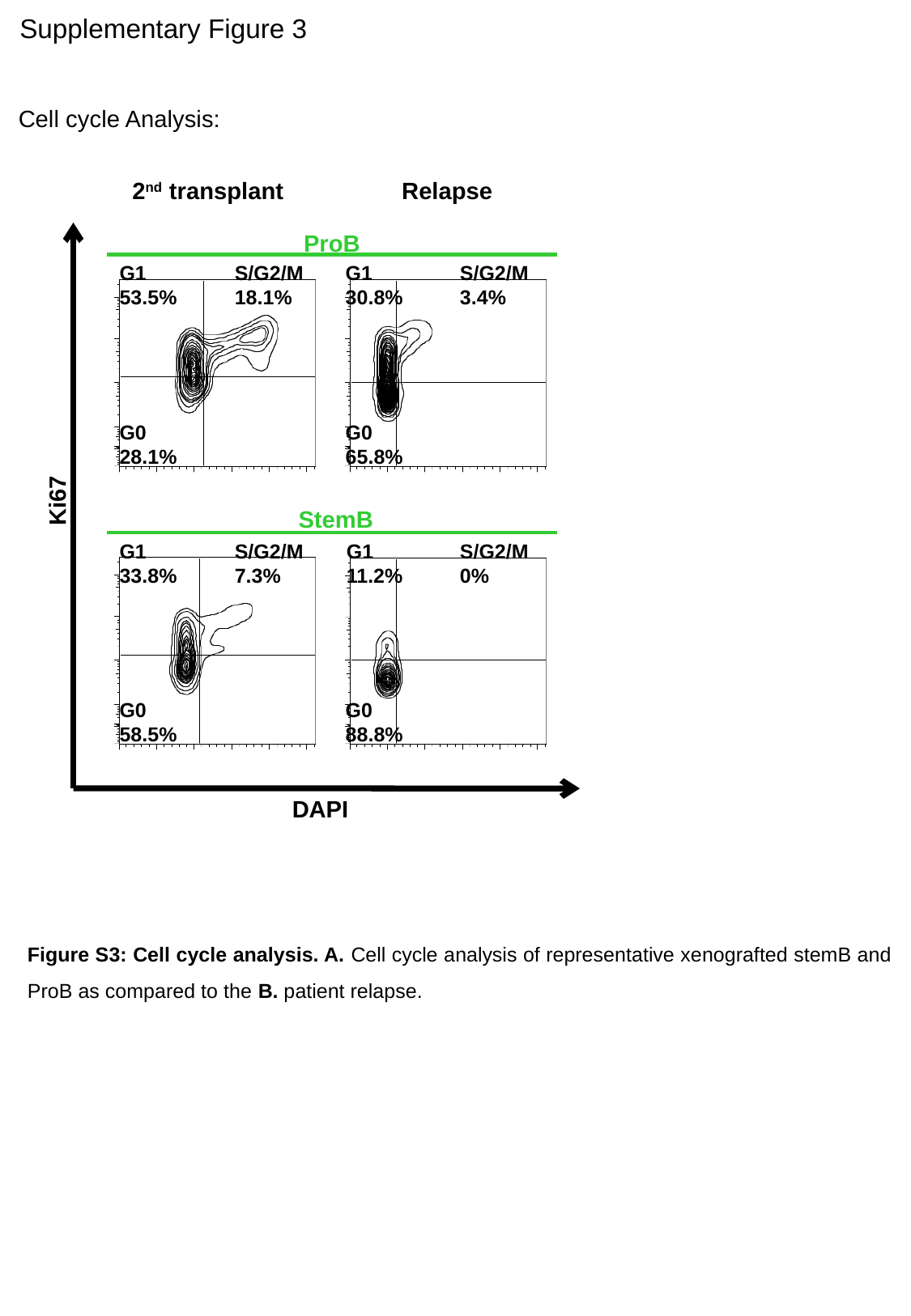

Supplementary Figure 3
Cell cycle Analysis:
Relapse
2nd transplant
ProB
G1
53.5%
S/G2/M
18.1%
G1
30.8%
S/G2/M
3.4%
G0
28.1%
G0
65.8%
Ki67
StemB
G1
33.8%
S/G2/M
7.3%
G1
11.2%
S/G2/M
0%
G0
58.5%
G0
88.8%
DAPI
Figure S3: Cell cycle analysis. A. Cell cycle analysis of representative xenografted stemB and ProB as compared to the B. patient relapse.

## Slide 4
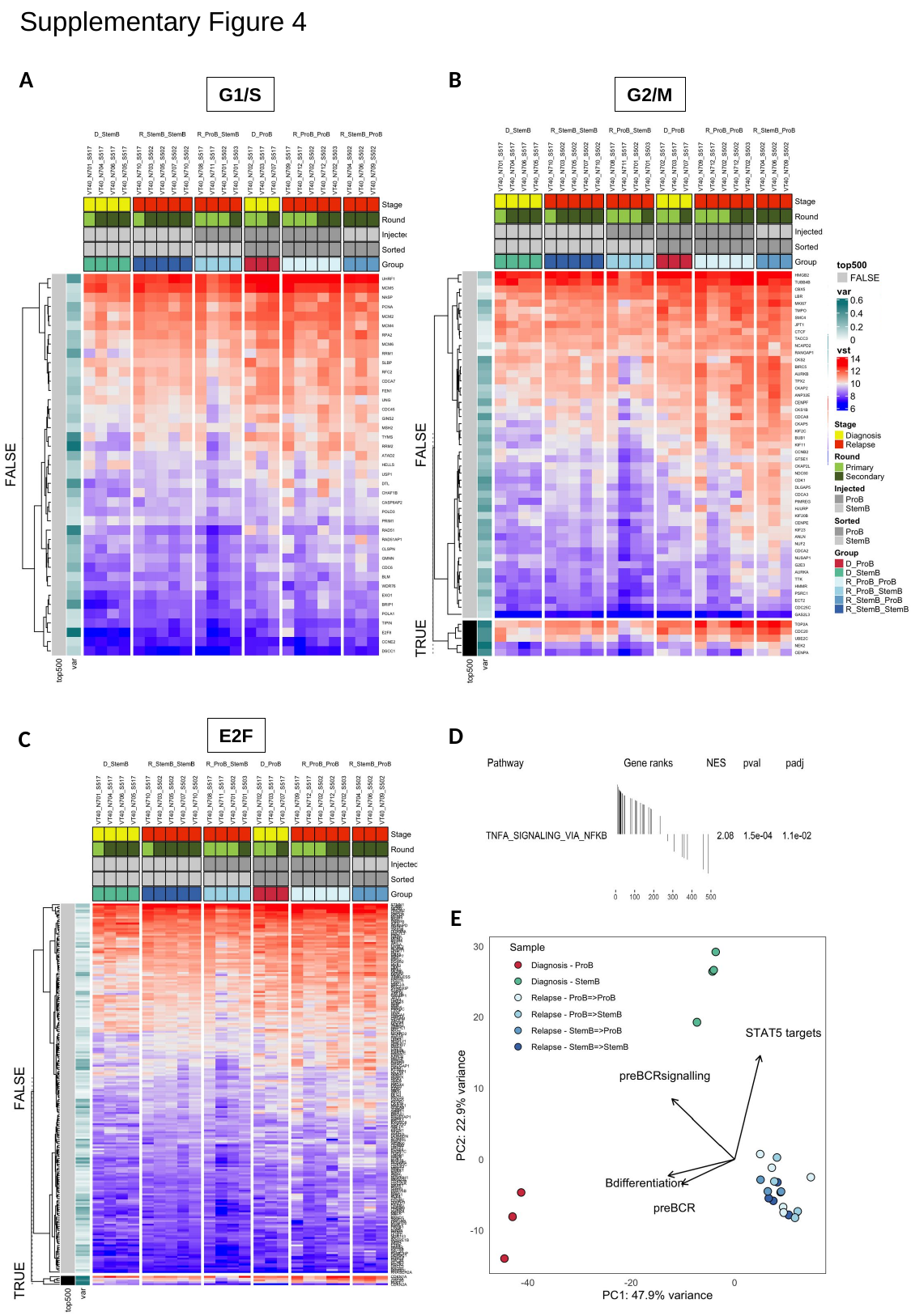

Supplementary Figure 4
A
B
G1/S
G2/M
D
C
E2F
E

## Slide 5
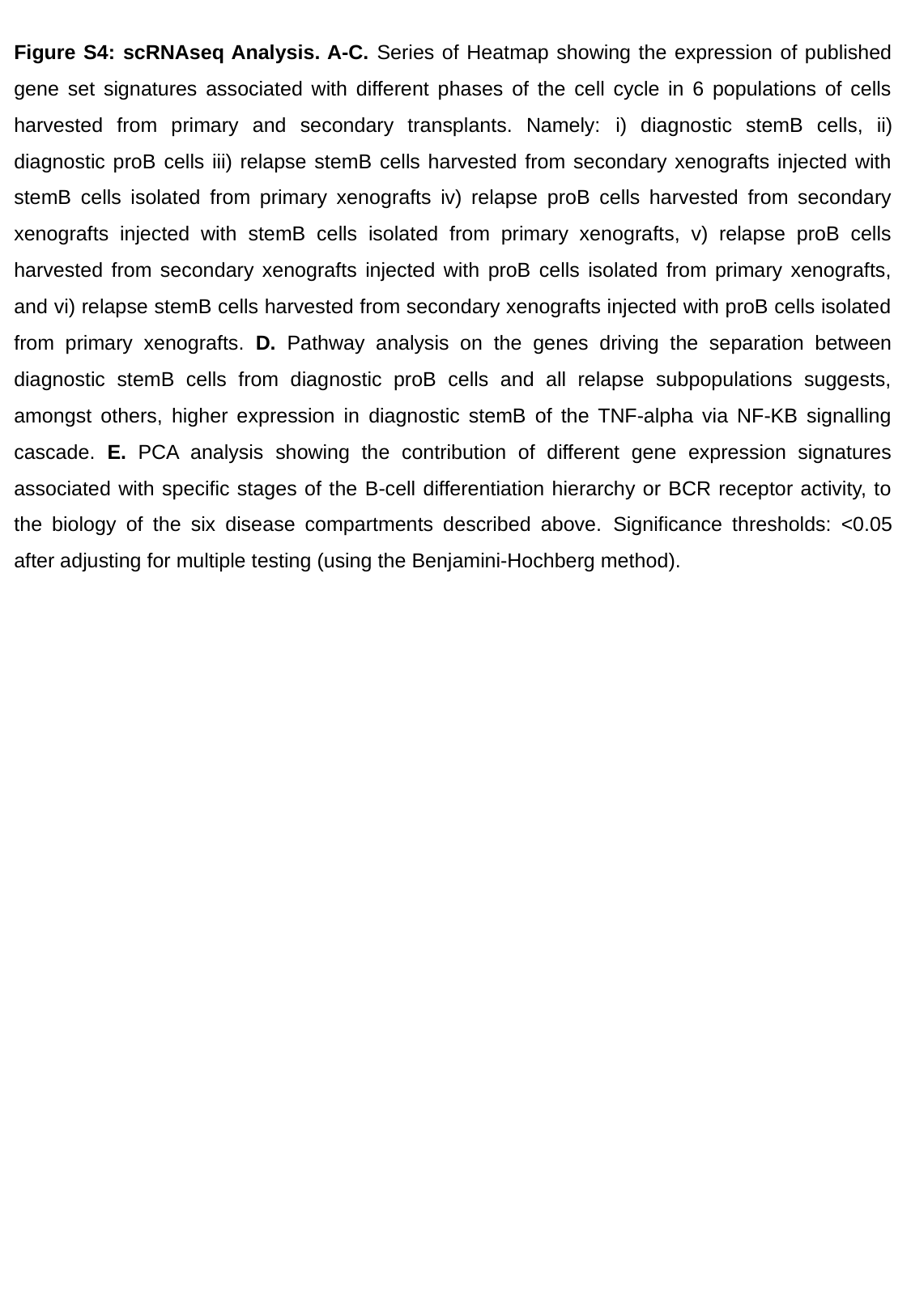

Figure S4: scRNAseq Analysis. A-C. Series of Heatmap showing the expression of published gene set signatures associated with different phases of the cell cycle in 6 populations of cells harvested from primary and secondary transplants. Namely: i) diagnostic stemB cells, ii) diagnostic proB cells iii) relapse stemB cells harvested from secondary xenografts injected with stemB cells isolated from primary xenografts iv) relapse proB cells harvested from secondary xenografts injected with stemB cells isolated from primary xenografts, v) relapse proB cells harvested from secondary xenografts injected with proB cells isolated from primary xenografts, and vi) relapse stemB cells harvested from secondary xenografts injected with proB cells isolated from primary xenografts. D. Pathway analysis on the genes driving the separation between diagnostic stemB cells from diagnostic proB cells and all relapse subpopulations suggests, amongst others, higher expression in diagnostic stemB of the TNF-alpha via NF-KB signalling cascade. E. PCA analysis showing the contribution of different gene expression signatures associated with specific stages of the B-cell differentiation hierarchy or BCR receptor activity, to the biology of the six disease compartments described above. Significance thresholds: <0.05 after adjusting for multiple testing (using the Benjamini-Hochberg method).

## Slide 6
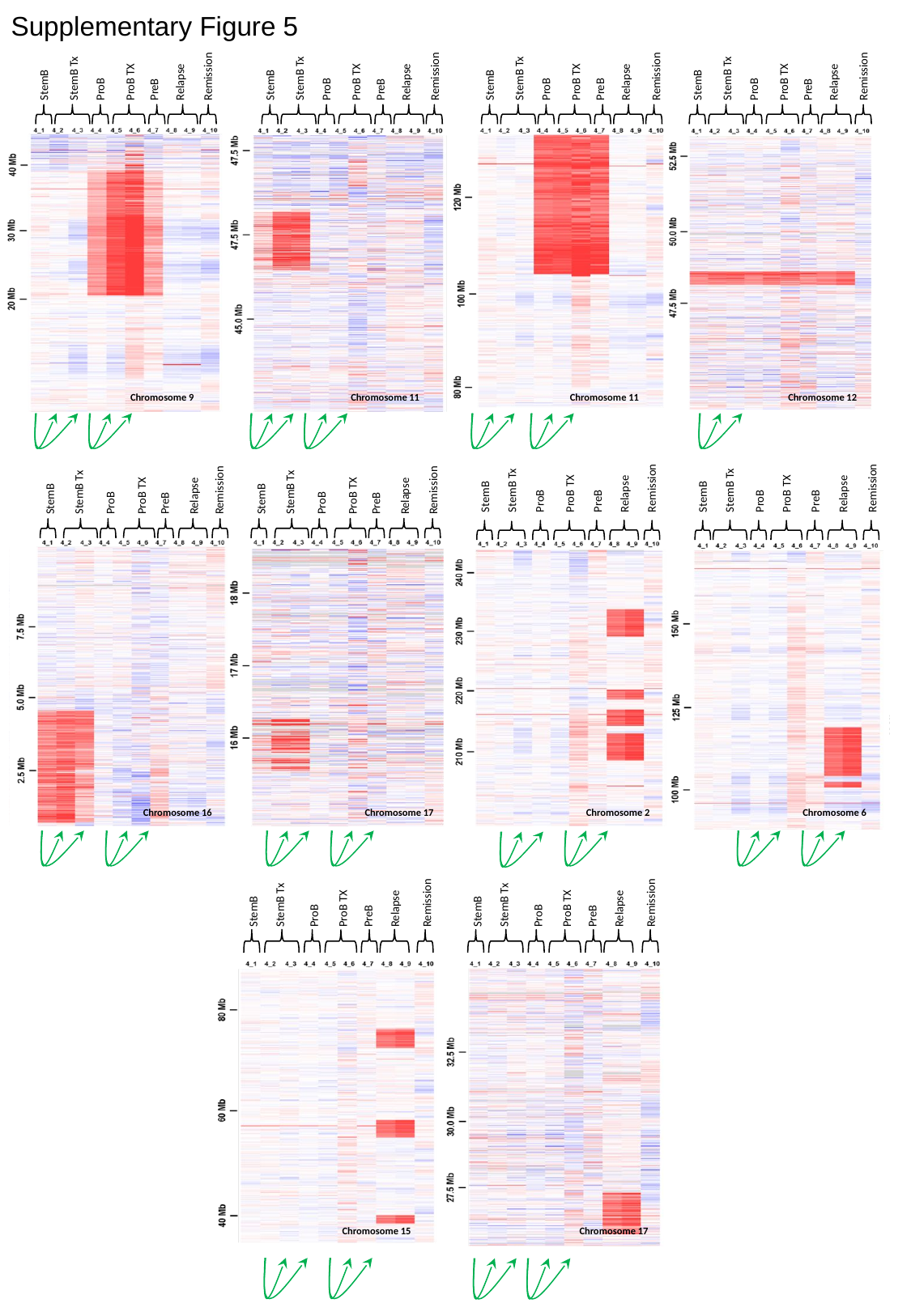

Supplementary Figure 5
Remission
Remission
Remission
Remission
StemB Tx
StemB Tx
StemB Tx
StemB Tx
StemB
ProB
ProB TX
PreB
Relapse
StemB
ProB
ProB TX
PreB
Relapse
StemB
ProB
ProB TX
PreB
Relapse
StemB
ProB
ProB TX
PreB
Relapse
Chromosome 9
Chromosome 11
Chromosome 11
Chromosome 12
StemB Tx
Remission
StemB Tx
Remission
StemB Tx
StemB Tx
Remission
Remission
ProB
ProB TX
PreB
Relapse
ProB
ProB TX
PreB
Relapse
StemB
StemB
StemB
ProB
ProB TX
PreB
Relapse
StemB
ProB
ProB TX
PreB
Relapse
Chromosome 16
Chromosome 17
Chromosome 2
Chromosome 6
StemB Tx
Remission
StemB Tx
Remission
StemB
ProB
ProB TX
PreB
Relapse
StemB
ProB
ProB TX
PreB
Relapse
Chromosome 15
Chromosome 17

## Slide 7
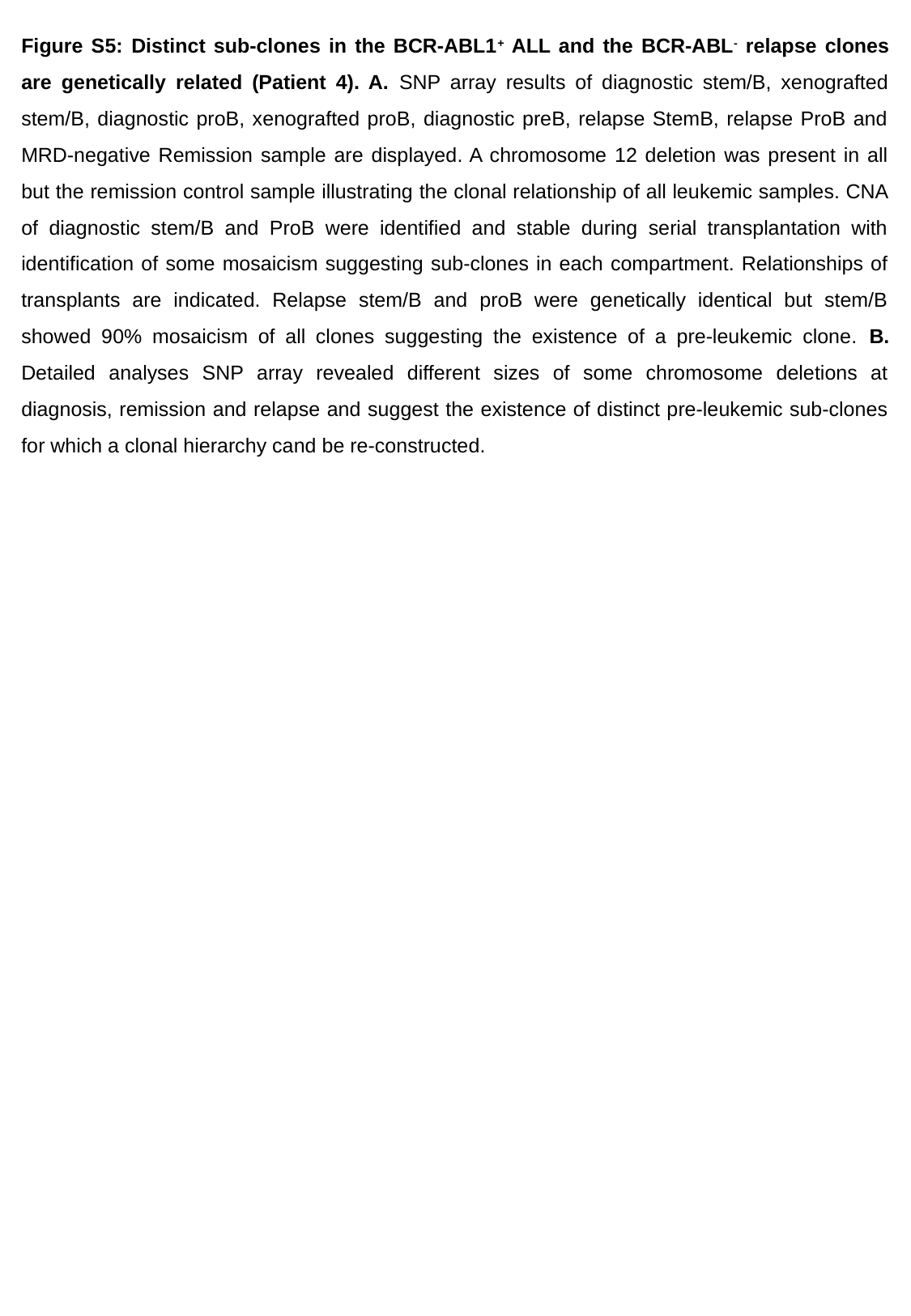

Figure S5: Distinct sub-clones in the BCR-ABL1+ ALL and the BCR-ABL- relapse clones are genetically related (Patient 4). A. SNP array results of diagnostic stem/B, xenografted stem/B, diagnostic proB, xenografted proB, diagnostic preB, relapse StemB, relapse ProB and MRD-negative Remission sample are displayed. A chromosome 12 deletion was present in all but the remission control sample illustrating the clonal relationship of all leukemic samples. CNA of diagnostic stem/B and ProB were identified and stable during serial transplantation with identification of some mosaicism suggesting sub-clones in each compartment. Relationships of transplants are indicated. Relapse stem/B and proB were genetically identical but stem/B showed 90% mosaicism of all clones suggesting the existence of a pre-leukemic clone. B. Detailed analyses SNP array revealed different sizes of some chromosome deletions at diagnosis, remission and relapse and suggest the existence of distinct pre-leukemic sub-clones for which a clonal hierarchy cand be re-constructed.
